# Knockdown of *FIBRILLIN4* Gene Expression in Apple Decreases Plastoglobule Plastoquinone Content

**DOI:** 10.1371/journal.pone.0047547

**Published:** 2012-10-12

**Authors:** Dharmendra K. Singh, Tatiana N. Laremore, Philip B. Smith, Siela N. Maximova, Timothy W. McNellis

**Affiliations:** 1 Department of Plant Pathology & Environmental Microbiology, The Pennsylvania State University, University Park, Pennsylvania, United States of America; 2 Intercollege Graduate Degree Program in Plant Biology, The Pennsylvania State University, University Park, Pennsylvania, United States of America; 3 The Huck Institutes for the Life Sciences, The Pennsylvania State University, University Park, Pennsylvania, United States of America; 4 Department of Horticulture, The Pennsylvania State University, University Park, Pennsylvania, United States of America; University of Massachusetts Medical, United States of America

## Abstract

Fibrillin4 (FBN4) is a protein component of plastoglobules, which are antioxidant-rich sub-compartments attached to the chloroplast thylakoid membranes. FBN4 is required for normal plant biotic and abiotic stress resistance, including bacterial pathogens, herbicide, high light intensity, and ozone; FBN4 is also required for the accumulation of osmiophilic material inside plastoglobules. In this study, the contribution of FBN4 to plastoglobule lipid composition was examined using cultivated apple trees in which *FBN4* gene expression was knocked down using RNA interference. Chloroplasts and plastoglobules were isolated from leaves of wild-type and *fbn4* knock-down trees. Total lipids were extracted from chloroplasts and plastoglobules separately, and analyzed using liquid chromatography-mass spectrometry (LC–MS). Three lipids were consistently present at lower levels in the plastoglobules from *fbn4* knock-down apple leaves compared to the wild-type as determined by LC-MS multiple ion monitoring. One of these species had a molecular mass and fragmentation pattern that identified it as plastoquinone, a known major component of plastoglobules. The plastoquinone level in *fbn4* knock-down plastoglobules was less than 10% of that in wild-type plastoglobules. In contrast, plastoquinone was present at similar levels in the lipid extracts of whole chloroplasts from leaves of wild-type and *fbn4* knock-down trees. These results suggest that the partitioning of plastoquinone between the plastoglobules and the rest of the chloroplast is disrupted in *fbn4* knock-down leaves. These results indicate that FBN4 is required for high-level accumulation of plastoquinone and some other lipids in the plastoglobule. The dramatic decrease in plastoquinone content in *fbn4* knock-down plastoglobules is consistent with the decreased plastoglobule osmiophilicity previously described for *fbn4* knock-down plastoglobules. Failure to accumulate the antioxidant plastoquinone in the *fbn4* knock-down plastoglobules might contribute to the increased stress sensitivity of *fbn4* knock-down trees.

## Introduction

Plastoglobules are lipoprotein structures found in chloroplasts, chromoplasts and other plastid types [1]. Plastoglobules are defined by a phospholipid monolayer and associated proteins surrounding a core of hydrophobic material [2], [3]. In chromoplasts, plastoglobules become enlarged and filled with carotenoid pigments, and contain several carotenoid biosynthetic enzymes [1]. In chloroplasts, plastoglobules are attached to thylakoid membranes [3]. Plastoglobules are probably formed from the thylakoid membrane by a blistering process [3], [4].

Plastoglobules have been found to contain a wide range of lipids, including plastoquinone, plastohydroquinone, phylloquinone K, α-tocopherol, α-tocoquinone, carotenoids, carotenoid esters, triacylglycerols, free fatty acids, glycolipids, and phospholipids [5]. Plastoglobule lipid constituents change with the developmental stage of the plant. Triacylglycerols decreased while carotenoids and carotenoid esters increased in plastoglobules during senescence in *Fagus sylvatica* (beech) [5]. Plastoglobule lipid composition varies between plant species, as well. For example, triacylglycerols and carotenoid esters are not detected or are present in very small amounts in young leaves of spinach and beech but are found in very high proportion in the plastoglobules of *Sarothamnus scoparius* [5]. Plastoglobules of *Vicia faba* (faba bean) chloroplasts contain α-tocopherol, plastoquinone, and triacylglycerols, and are devoid of carotenoids and chlorophyll [6]. Plastoglobules of *Beta vulgaris* (sugar beet) contain chlorophyll but no β-carotene [7], [8].

Some types of lipids found in plastoglobules are involved in photosynthesis and reactive oxygen species (ROS) scavenging. Phylloquinone and plastoquinone are components of the electron transport system in chloroplasts [9]. Plastoquinone amount increases during stress [10]. Along with α-tocopherol, plastoquinone scavenges ROS generated at photosystem II (PSII) during high-light stress in *Chlamydomonas reinhardtii* [11]. Plastoquinone has been shown to protect the D1 and D2 reaction center proteins of PSII [11]. A study using a tocopherol synthesis enzyme mutant suggested that tocopherol protects PSII from photoinactivation [12]. Tocopherols are also important membrane lipid peroxidation inhibitors and scavengers of ROS in the chloroplast [13], [11].

**Figure 1:**
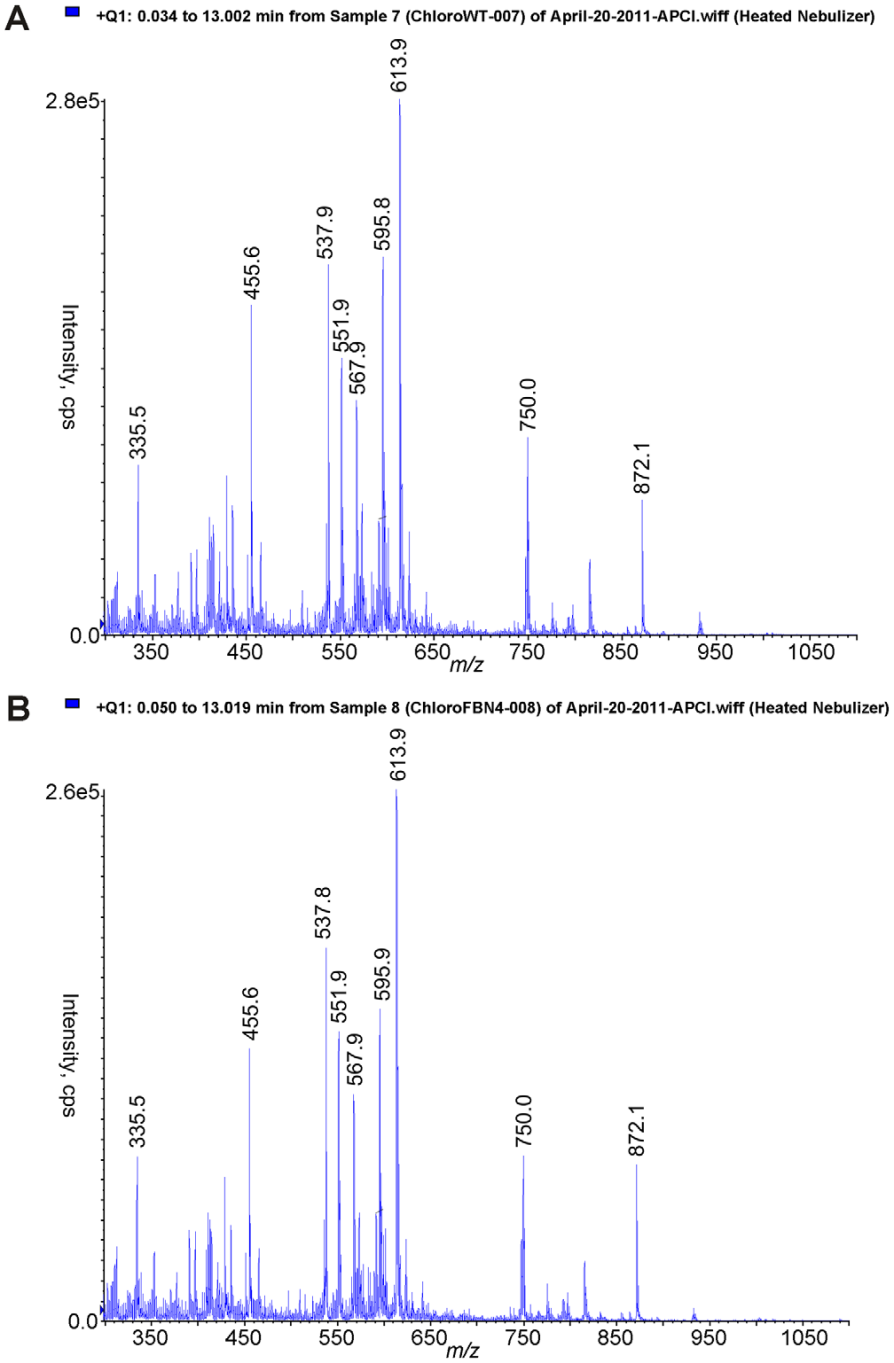
Full MS scans of WT (A) and *fbn4*. ** KD (B) chloroplast lipid extracts show similar compositions of the two samples.** Figure shows raw data that have not been normalized to retinol.

**Figure 2:**
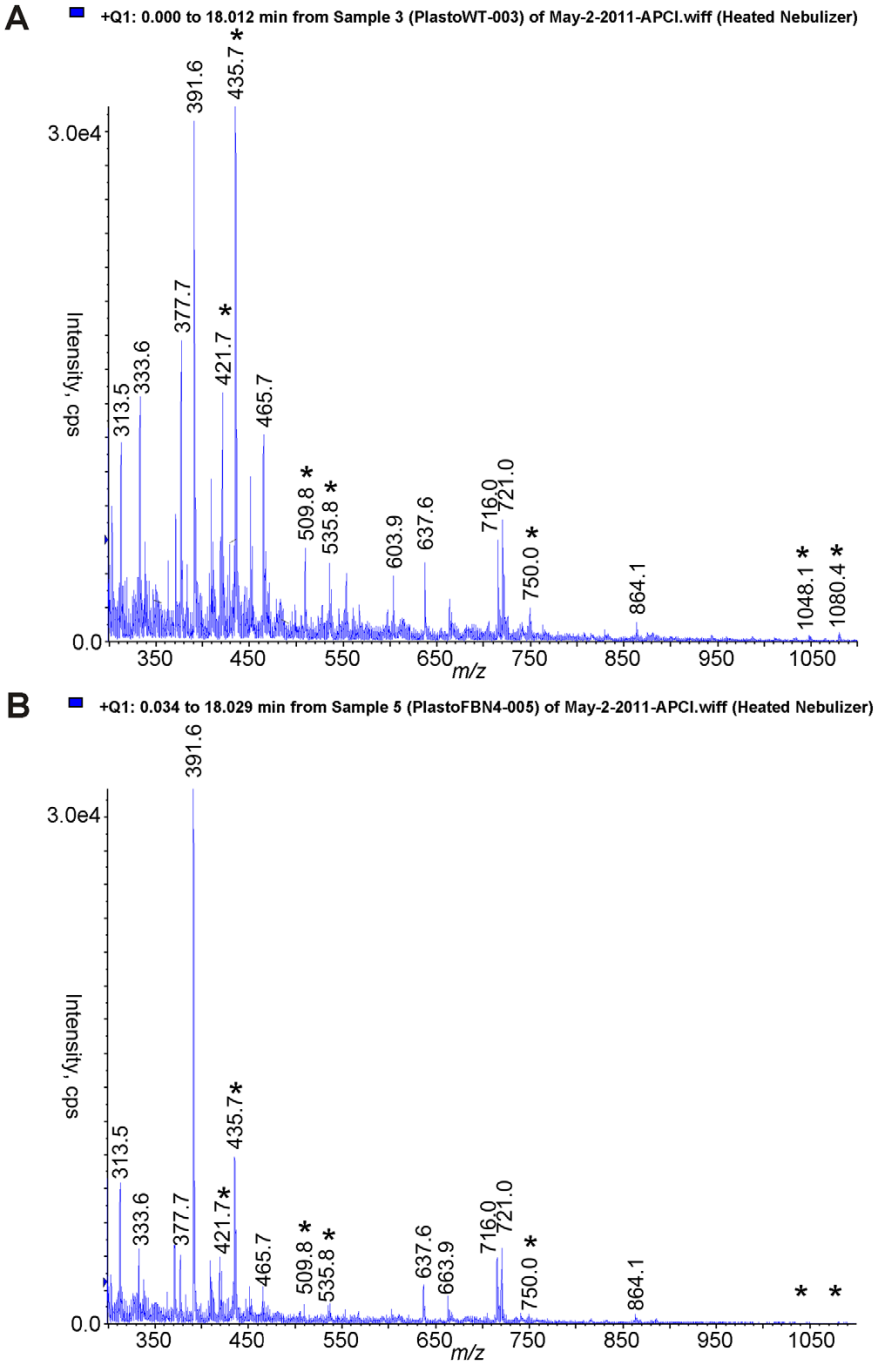
Full MS scans of WT (A) and *fbn4* KD (B) plastoglobule lipid extracts show some differences between samples. Asterisks indicate species with differences between panels (A) and (B). Figure shows raw data that have not been normalized to retinol.

**Table 1:** Selected analytes that were more abundant in *fbn4* KD plastoglobules compared to wild-type plastoglobules in a full-scan LC-MS.

Analyte *m/z*	WT:*fbn4* KD analyte abundance ratio
421	36
435	527
509	43
535	39
537	17
749	117
1048	44
1080	>1000

Plastoglobules may play roles in plant development and stress tolerance. In broad bean and rhododendron, significantly larger plastoglobules were observed in older leaves than in younger leaves [4]. During the chloroplast to chromoplast transition, plastoglobules enlarge and accumulate carotenoids [4]. Enlargement of plastoglobules was observed during ozone treatment in aspen and spruce trees [4]. Plastoglobule size also increases during drought [14] and in plants growing in the presence of heavy metals [15], [16].

**Figure 3:**
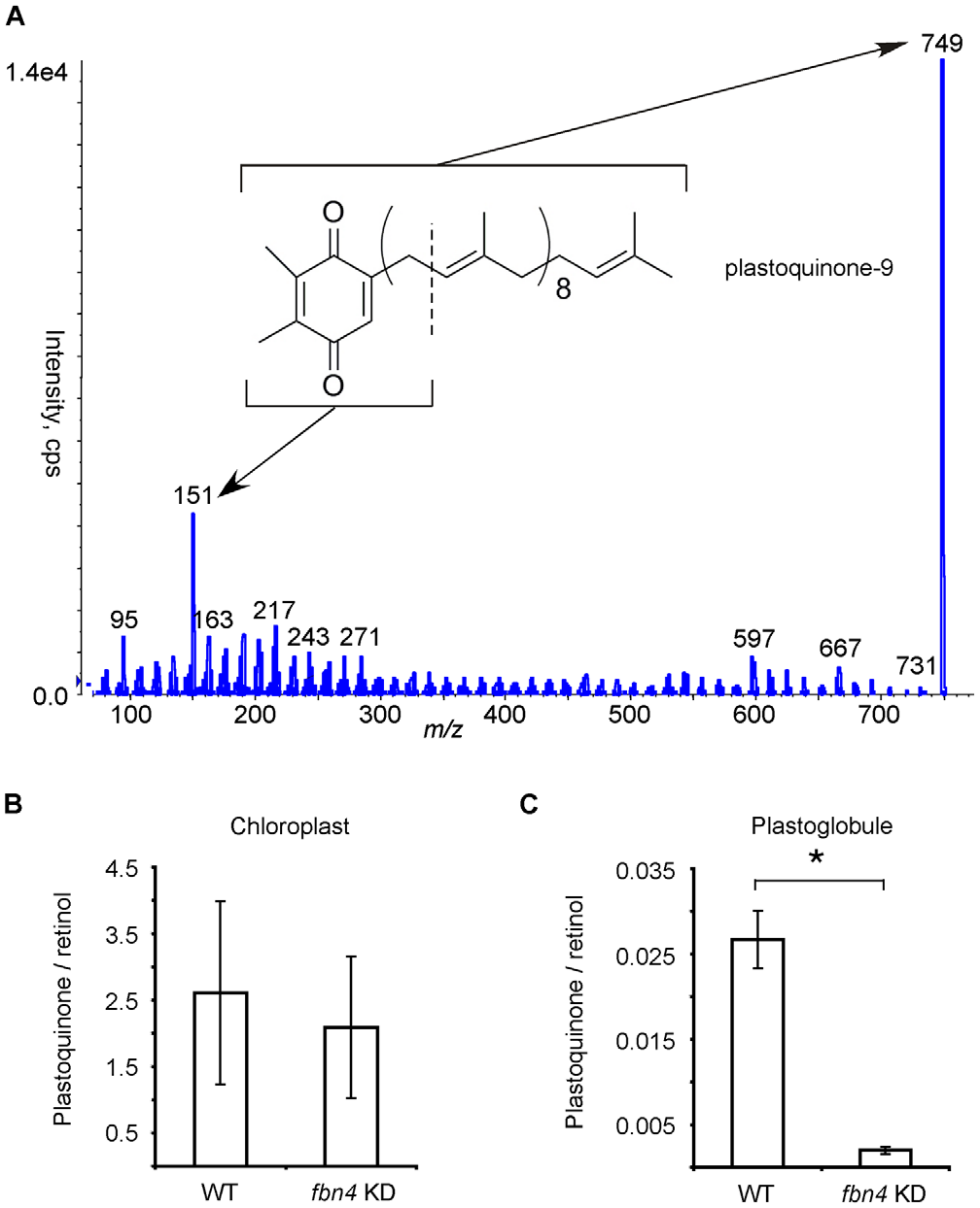
Analyte at m/z 749. (A) MS/MS analysis and possible structure. Abundance in WT and *fbn4* KD lipid extracts from chloroplasts (B) and plastoglobules (C), normalized to retinol. Data are means ± SD of three measurements; *, *P*<0.05 using Student's *t* test. Similar results were obtained in two biological replicates for the chloroplasts and three biological replicates for the plastoglobules.

The fibrillins are a highly conserved protein family linked to plant stress tolerance and plastoglobule structural maintenance (reviewed in [17]). Fibrillins from algae and plants can be divided in 12 sub-families (reviewed in [17]). Plastoglobules can be formed *in vitro* from carotenoids and pepper fibrillin (FBN1) protein [2]. Overexpressing bell pepper fibrillin (FBN1) in tomato and tobacco resulted in plastoglobule clustering, suggesting fibrillin involvement in plastoglobule formation [18], [19]. *fbn4* knock-down (*fbn4* KD) apple trees exhibited no changes in plastoglobule number, but did exhibit a sharply decreased number of osmiophilic plastoglobules compared to the wild-type (WT) [20]. Plastoglobule osmiophilicity could be due to the presence of unsaturated lipids [20], and therefore the reduced osmiophilicity of *fbn4* KD plastoglobules suggests that they have different lipid content than WT plastoglobules.

**Figure 4:**
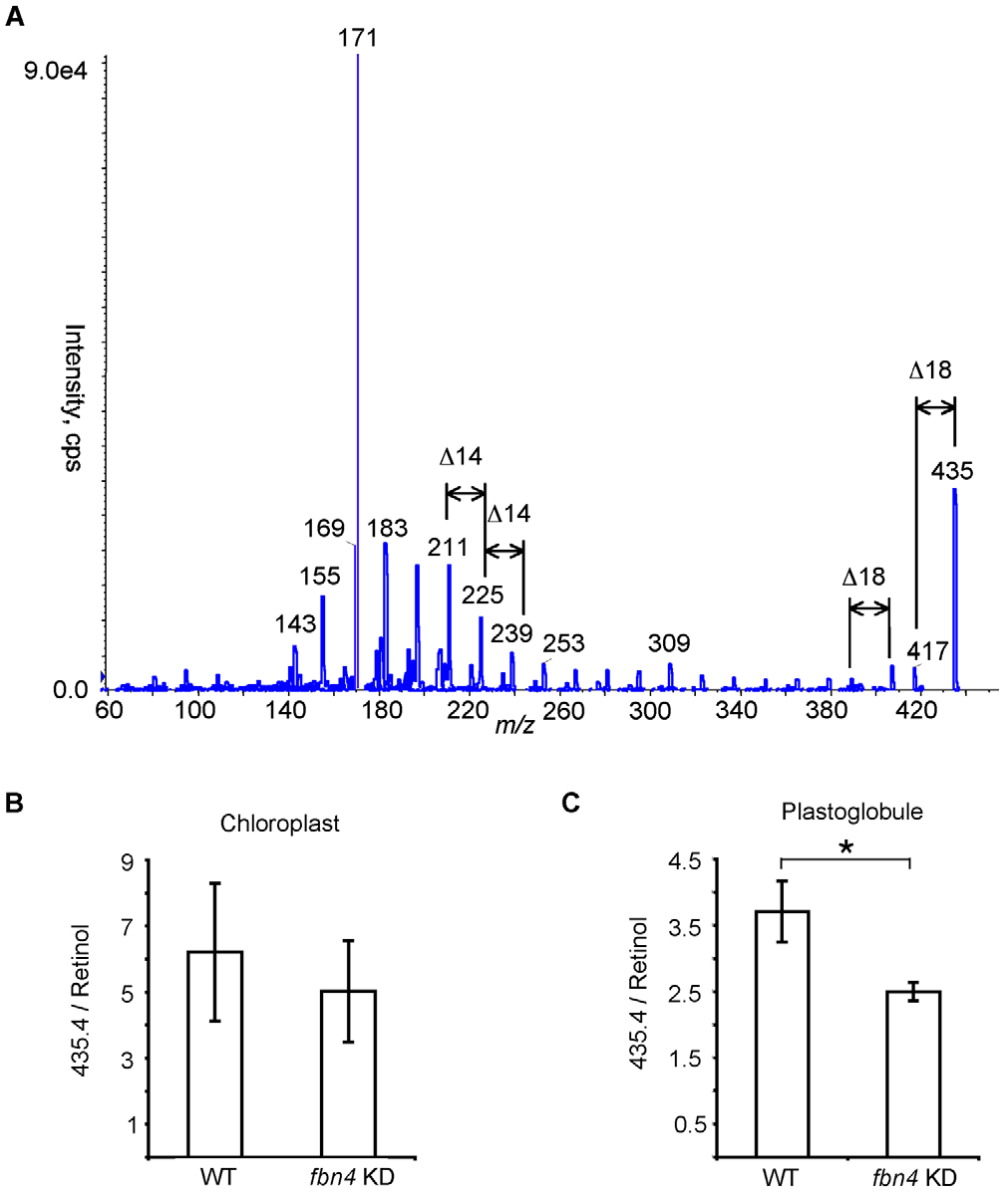
Analyte at *m/z* 435. (A) MS/MS analysis. Abundance in WT and *fbn4* KD lipid extracts from chloroplasts (B) and plastoglobules (C), normalized to retinol. Data are means ± SD of three measurements; *, *P*<0.05 using Student's *t* test. Similar results were obtained in two biological replicates for the chloroplasts and three biological replicates for the plastoglobules.

Lipids have been classified into eight categories by the LIPID MAPS consortium [21]. Plastoglobules contain lipids representing at least three of these categories: 1) the prenol lipids plastoquinone, α-tocopherol, plastohydroquinone, phylloquinone K, carotenoids; 2) glycerolipids triacylglycerols; and 3) free fatty acids from the fatty acyl category. In addition, plastoglobules contain glycolipids and phospholipids [5], [22], which could not be grouped into any lipids categories because of insufficient information about these lipids in plastoglobules. Lipids encompass a very diverse group of compounds, and despite recent advances in lipidomic analysis, not all lipid categories can be analyzed using a single MS technique because of their different physicochemical properties resulting from the presence of various functional groups [23] (reviewed in [24]). Atmospheric pressure chemical ionization liquid chromatography-mass spectrometry (APCI LC-MS) in positive ion mode was used in this study, because the major plastoglobule lipids such as prenol lipids and glycerolipids are amenable to analysis by this technique. In this study, chloroplast and plastoglobule lipids from leaves of WT and *fbn4* KD trees were analyzed to determine the effects of decreased FBN4 protein on plastoglobule content.

**Figure 5:**
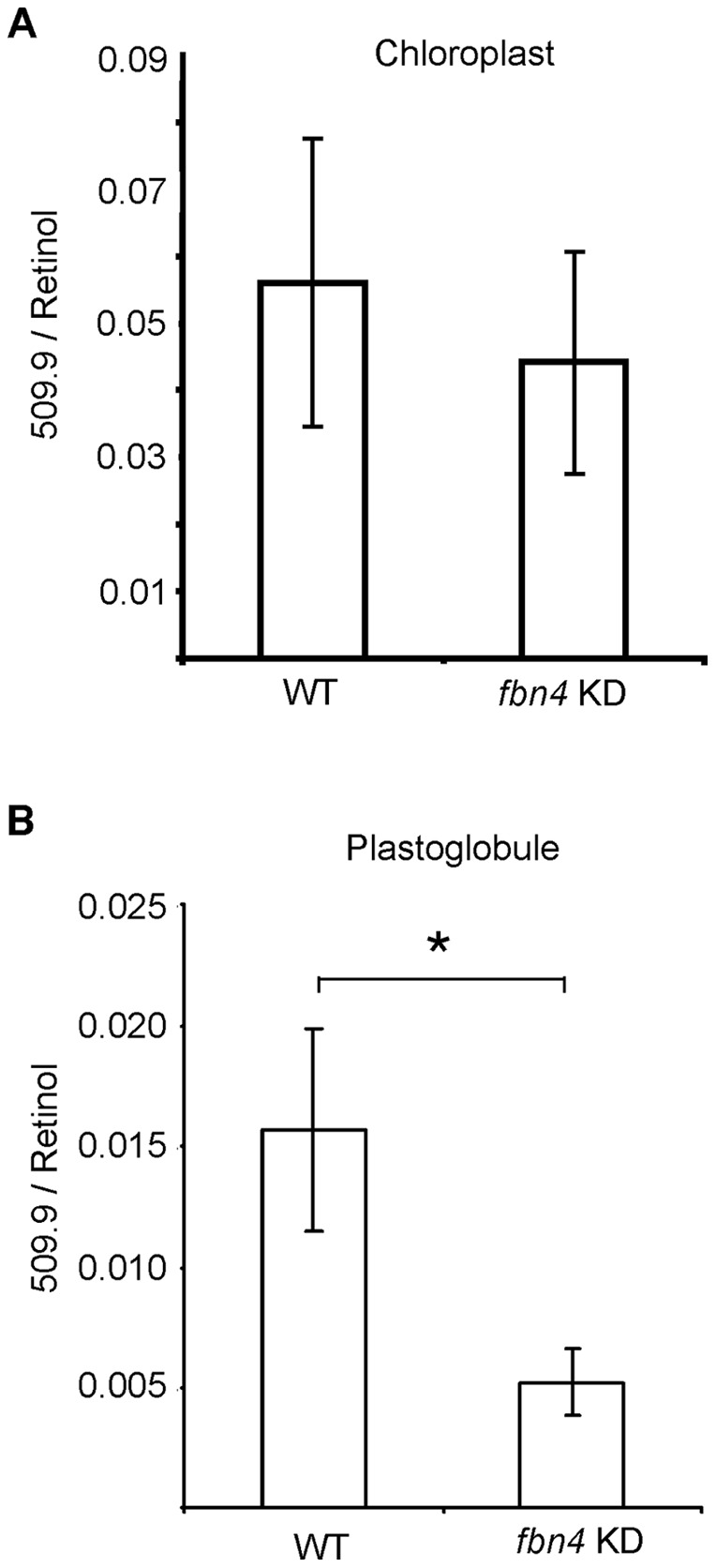
Analyte at *m/z* 510. Abundance in WT and *fbn4* KD lipid extracts from chloroplasts (B) and plastoglobules (C), normalized to retinol. Data are means ± SD of three measurements; *, *P*<0.05 using Student's *t* test. Similar results were obtained in two biological replicates for the chloroplasts and three biological replicates for the plastoglobules.

## Results

### Full-scan LC-MS of chloroplast and plastoglobule lipid content

Chloroplasts and plastoglobules were isolated from leaves of WT and *fbn4* KD apple trees. Full-scan mass spectra were used for comparison of relative lipid levels in plastoglobules of WT and *fbn4* KD apple trees. A known amount of retinol was added to each chloroplast or plastoglobule sample prior to lipid extraction and the instrument response was normalized to the signal at *m/z* 285 when comparing lipid profiles between different samples. Retinol was selected for this purpose because it has similar physicochemical properties to the analytes of interest. Our preliminary experiments determined that it was not present in plastoglobule and chloroplasts extracts from apple leaves.

**Figure 6:**
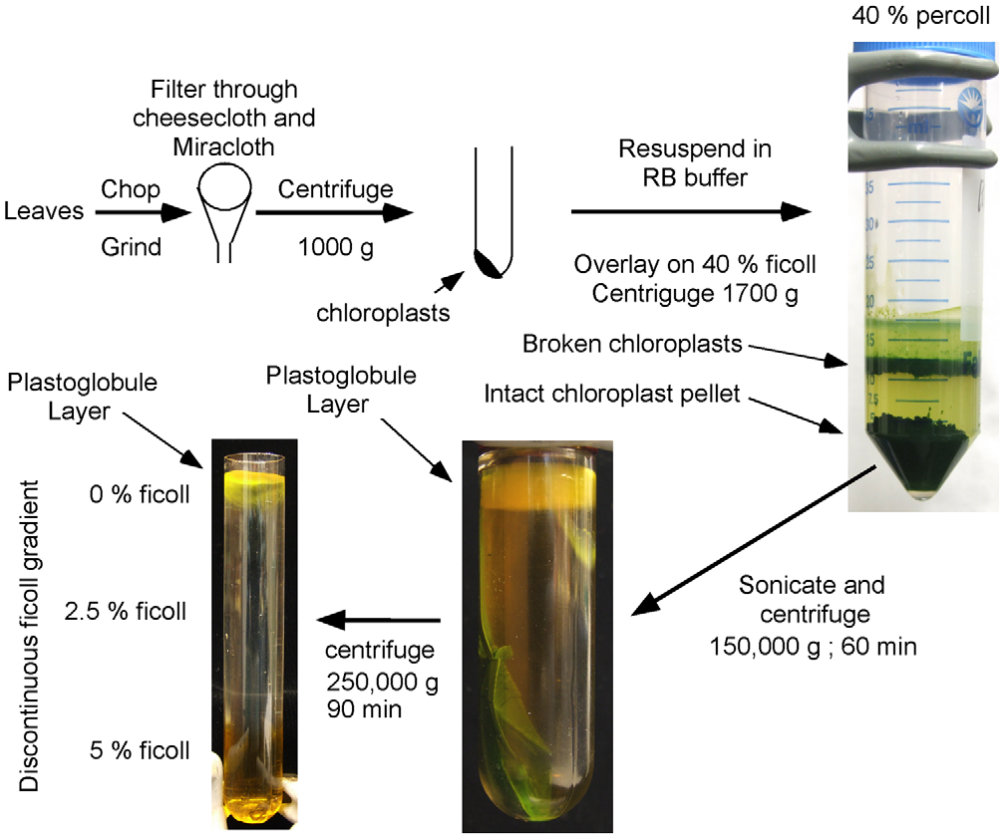
Schematic representation of chloroplast and plastoglobule extraction process from apple leaves.

Full-scan LC-MS analyses indicated no major differences in lipid composition of chloroplasts from WT and *fbn4* KD apple trees (Fig. 1). As expected, apple plastoglobules contained a complex mixture of lipids, with the relative levels of most of lipids being similar in WT and *fbn4* KD (Fig. 2). We focused our search on molecules that might be missing or at lower levels in *fbn4* KD plastoglobules compared to WT plastoglobules. Seven lipids that were at least 35 times more abundant in the WT than in *fbn4* KD apple leaf plastoglobules (Table 1) were selected for LC-MS multiple ion monitoring (MIM). The 35-fold difference cutoff was arbitrarily selected. Limiting the number of ions selected for the MIM experiments improves analytical sensitivity. An additional peak at *m/z* 537, which was 17-fold more abundant in the WT samples compared with the *fbn4* KD samples, was selected for the MIM experiments because its LC retention time and MW were consistent with those of carotene, an antioxidant. MIM analyses revealed that only three of the eight analytes were consistently less abundant in *fbn4* KD plastoglobules compared to WT plastoglobules in three independent biological replicates.

**Figure 7:**
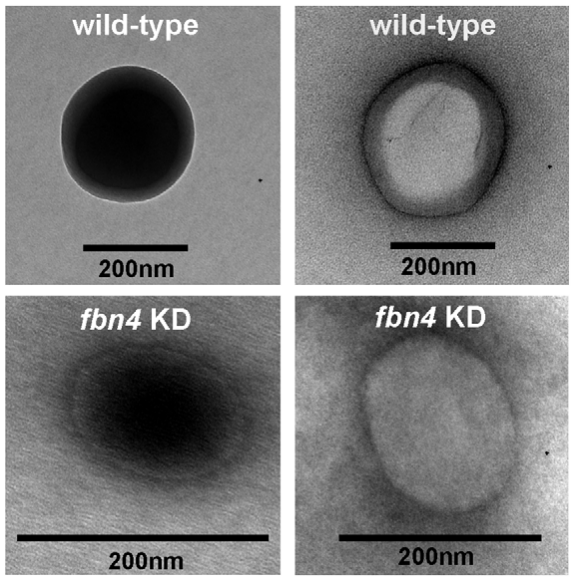
Transmission electron micrographs of extracted plastoglobules of WT and *fbn4* KD apple tree leaves. Leaves were harvested from 4 to 5 month-old trees grown under 90 *µ*E m^−2^ s^−1^ light intensity with a 12 h photoperiod. Extracted plastoglobules were stained with OsO_4_ and observed on a copper mesh support. Transmission electron micrographs of electron opaque (black) and electron transparent (white) plastoglobules extracted from WT (upper row) and *fbn4* KD (lower row) are shown.

### Analyte at *m/z* 749

Positive-ion LC-MS/MS was used for characterization of the ion at *m/z* 749, which most likely corresponded to the protonated molecular ion for plastoquinone (MW 748.62; C_53_H_80_O_2_), as shown in Figure 3A. The ion at *m/z* 749was mass selected in the first quadrupole, fragmented by collision-induced dissociation, and the resulting product ion mass spectrum (Fig. 3A). The peak at *m/z* 151 had the mass expected for a fragment of plastoquinone containing the quinone ring (Fig. 3A). A similar fragmentation pattern involving loss of the isoprenyl side chain was observed in LC-MS/MS analysis of coenzyme Q, which is a structurally similar to plastoquinone [25], [26].

The abundance of the species detected at *m/z* 749 was compared in chloroplasts and plastoglobules of WT and *fbn4* KD apple tree leaves using LC-MS with MIM. The 749 *m/z* signal was normalized to that of retinol, which was added to the samples prior to the chloroplast and plastoglobule lipid extractions as a surrogate and was used to normalize differences in extraction efficiency and sample processing. The observed abundance of 749 *m/z* ion in WT and *fbn4* KD chloroplast extracts was similar (Fig. 3B). However, 749 *m/z* ion was approximately 13 times more abundant in the WT plastoglobule extracts compared to *fbn4* KD plastoglobule extracts (Fig. 3C). These results suggest that the species at *m/z* 749 is less abundant in plastoglobules from the leaves of *fbn4* KD trees compared to the WT plastoglobules, while its abundance in whole chloroplasts is similar in both genotypes.

### Analyte at *m/z* 435

Inspection of the full-scan LC-MS profiles indicated that the ion at *m/z* 435 was over 500-fold more abundant in WT plastoglobule extracts compared to *fbn4* KD plastoglobule extracts (Table 1). The LC-MS/MS product ion mass spectrum of this ion suggested that the analyte contained at least one oxygen atom, because two pairs of fragments had a difference in mass of 18 Da, corresponding to a loss of water (*m/z* 435 and *m/z* 417; and *m/z* 407 and *m/z* 389; Fig. 4A). Fragmentation pattern of the 435 *m/z* ion indicates the presence of an alkyl chain, because its product ion mass spectrum contained a number of fragments separated by 14 Da (CH_2_). However, it is impossible to draw a single structure of this analyte based on the molecular weight and fragmentation pattern using LC-MS/MS.

The abundance of the species detected at *m/z* 435 was compared in chloroplasts and plastoglobules from leaves of WT and *fbn4* KD apple trees using MIM. Levels of the species at 435 *m/z*, normalized to retinol, were similar in lipid extracts from whole chloroplasts of WT and *fbn4* KD apple leaves (Fig. 4B). However, the analyte at 435 *m/z* was approximately 1.5 times more abundant in WT plastoglobule extracts compared to *fbn4* KD plastoglobule extracts (Fig. 4C). These results suggest that *m/z* 435 ion was less abundant in *fbn4* KD plastoglobules compared to WT plastoglobules, while its abundance in whole chloroplasts was comparable between *fbn4* KD and WT trees.

### Analyte at *m/z* 510

The full-scan LC-MS analysis revealed that analyte at *m/z* 510 was more abundant in the plastoglobules of WT than the plastoglobules of *fbn4* KD apple leaves (Table 1). According to LC-MS MIM analysis, the species with *m/z* 510 was present in similar amounts in lipid extracts of whole chloroplasts from WT and *fbn4* KD apple tree leaves (Fig. 5A). In contrast, the species with *m/z* 510 was three times more abundant in the WT plastoglobule extracts compared to *fbn4* KD plastoglobule extracts (Fig. 5B). These results suggest that the species at *m/z* 510 was less abundant in plastoglobules in *fbn4* KD trees compared to plastoglobules in WT trees, while the abundance of the species with *m/z* 510 in whole chloroplasts is comparable between *fbn4* KD and WT trees. Identification of the structure of this compound by tandem MS analysis of the 510 *m/z* ion was unsuccessful.

## Discussion

Plastoglobule osmiophilicity is sharply decreased in *fbn4* KD apple leaf chloroplasts compared to the wild-type, suggesting that plastoglobule contents are altered in *fbn4* KD plants [20]. The present study supports this hypothesis, because several compounds were found to be consistently present at reduced levels in *fbn4* KD chloroplast plastoglobules compared to the wild-type. More specifically, the decreased plastoglobule osmiophilicity in *fbn4* KD plants suggests that plastoglobules in *fbn4* KD chloroplasts have decreased reducing agent content [20]. The analyses performed in the present study supported this hypothesis by revealing a >90% reduction in a species with a molecular mass and molecular fragmentation pattern consistent with plastoquinone (*m/z* 749). Plastoquinone has been found to be a major constituent of plastoglobules [27], [28]. Plastoquinone is a strong antioxidant [29], [30], [11] with nine ethylenic bonds, which can reduce OsO_4_ to form stable diester adducts [31] that are opaque to electrons in TEM due to the high mass density of osmium [32]. Lower levels of plastoquinone, and possibly the analytes at *m/z* 435 and *m/z* 510, in the plastoglobules of *fbn4* KD chloroplasts compared to the wild-type might account for the reduced osmiophilicity of *fbn4* KD chloroplast plastoglobules in TEM [20]. Apple *fbn4* KD trees and *fbn4* mutant *Arabidopsis thaliana* plants are sensitive to a number of abiotic and biotic plant stresses, including ozone, bacterial pathogens, high light intensity and herbicide [20]. The results presented here suggest that FBN4-dependent accumulation of plastoquinone and possibly other compounds in the plastoglobule is required for normal stress tolerance.

Interestingly, levels of plastoquinone were similar in lipid extracts of WT and *fbn4* KD leaf chloroplasts, despite the >90% decrease in levels of plastoquinone in *fbn4* KD plastoglobules compared to WT plastoglobules. This suggests that the partitioning of plastoquinone between the plastoglobule and the rest of the chloroplast is altered in *fbn4* KD plants. Depending on the plant species and environmental conditions, from 20% to 75% of chloroplast plastoquinone is normally present in the plastoglobules [27], [28]. Plastoquinone may fail to accumulate to high levels in the plastoglobules of *fbn4* KD leaf chloroplasts. This would most likely be due to reduced plastoquinone movement into the plastoglobules, since plastoquinone is synthesized on the thylakoid and chloroplast membranes [33], and no known plastoquinone biosynthesis enzymes have been detected in plastoglobules [13], [8].

These results suggest that FBN4 protein plays a role in the accumulation of plastoquinone, as well as the analytes at *m/z* 510 and *m/z* 435, in chloroplast plastoglobules. The presence of a highly conserved lipocalin domain (reviewed in [34], [35]) in the FBN4 protein implies that FBN4 might bind and transport small hydrophobic molecules such as lipids and steroids [36], [20]. It is possible that FBN4 is involved in the transport of plastoquinone and other molecules from thylakoids into the plastoglobules.

It should be noted that this study was not expected to detect all differences in metabolite levels between wild-type chloroplasts and plastoglobules, and some differences may have been missed. The full-scan LC-MS analyses provided broad coverage of relative level of lipids in the samples; however, ability to accurately quantify individual lipids using full-scan LC-MS was compromised by the complexity of the mixture. Candidate molecules that appeared to be present in different amounts in WT and *fbn4* KD plastoglobules were selected based on the full-scan LC-MS data. These specific molecules were quantified accurately using LC-MS MIM. Targeted analysis of selected ions using MIM reduces noise, thereby improving data quality and increasing sensitivity. The fact that only three of the candidate molecules selected from the full-scan LC-MS data was consistently present at different levels in WT and *fbn4* KD plastoglobules is likely due to the noise present in the full-scan LC-MS data. For these reasons, this study should be considered a partial characterization of lipid differences between WT and *fbn4* KD apple leaf plastoglobules and chloroplasts.

The multiple *fbn4* KD lines previously generated had a similar stress-sensitive phenotype, as did *Arabidopsis thaliana* mutants of the orthologous *FBN4* gene [20]; due to plant production limitations and the large number of trees needed to complete the present study, a single representative *fbn4* KD apple line was selected and used for this project. The transgenic line used for this study was also used in the previous study and has a representative *fbn4* KD phenotype [20]. Nevertheless, the use of a single transgenic line is a limitation of the present study.

## Materials and Methods

### Plant material

Apple (*Malus* x *domestica*) plant growth conditions and development of the *fbn4* KD apple lines was described previously [20]. Rooted apple plants were grown in a greenhouse in potting mix (Redi-Earth) with a 10-h photoperiod under 90 μE m^−2^ s^−1^ light intensity.

### Isolation of intact chloroplasts and plastoglobules

Mature, green apple leaves (100 g) were washed and homogenized as described [37]. The chloroplast suspension was loaded on a 40% percoll cushion and centrifuged at 2,500 g at 4°C for 10 min in a swing-out rotor in a Sorvall RT7 centrifuge. The pellet contained intact chloroplasts. Plastoglobules were isolated from the intact chloroplasts using an established protocol (Fig. 6) [22]. In brief, the chloroplasts pellets were suspended in 1 mM phosphate buffer (pH 7.8) with 0.1 mM dithioerythritol and subsequently sonicated using Branson Sonifier S-450A set at duty cycle 50% and output control 2 for 7 min, followed by a 60-min centrifugation at 150,000 g using a Beckman L8-M ultracentrifuge equipped with a Ti70 rotor. The resulting yellow turbid zone near top of the tubes (Fig. 6) was collected and ficoll powder was added to make a 5% ficoll solution. A discontinuous gradient was prepared by placing the plastoglobule suspension in 5% ficoll at the bottom of a centrifugation tube, overlaying it with 2.5% ficoll and then with phosphate buffer (1 mM) containing no ficoll. After a 90-min centrifugation at 250,000 g in a Beckman SW-41Ti rotor, plastoglobules were collected from top of the tube (Fig. 6). The pellets were re-extracted once using the same method and the extracted plastoglobules were pooled for lipid analysis.

### Transmission electron microscopy of extracted plastoglobules

Extracted plastoglobules from WT and *fbn4* KD apple tree were stained with 0.5 % OsO_4_ and placed on a 200 mesh formvar-coated copper grid, stabilized with carbon film. Plastoglobules were observed with a JEOL 1200 EX II transmission electron microscope (TEM) fitted with high-resolution Tietz F224 digital camera. The presence of plastoglobules in extracts from WT and *fbn4* KD apple trees was confirmed by TEM imaging (Fig. 7). Both osmiophilic and non-osmiophilic plastoglobules were observed in extracts from WT and *fbn4* KD apple trees (Fig. 7).

### Chloroplast lipid extraction

An amount of chloroplasts containing 0.3 mg of total chlorophyll were used for each lipid extraction. Total chlorophyll was determined using the method of Arnon [38]. Lipids were extracted from chloroplasts by vortexing in cold acetone, followed by a brief sonication (10 s pulse), and then incubated in the dark at room temperature for 1 h. The sample was then centrifuged at 13,000 g for 1 min and the supernatant was collected. The pellet was re-extracted using the same method two more times. The supernatants were pooled and filtered through a 0.2 μM pore size PTFE membrane (Whatman International Ltd., Maldstone, UK).

### Plastoglobule lipid extraction

One third of the purified plastoglobule fraction was used for protein quantification in the sample. Proteins from the plastoglobules were extracted using an established protocol [8]. In brief, plastoglobule proteins were precipitated for 16 h with 100% acetone at −20°C. The precipitate was incubated in 0.2% acetic acid/10% methanol/and 80% acetone (v/v/v) for 30 min at −20°C. After centrifugation, supernatants were removed and the pellets were dissolved in DMSO and quantified by Bradford assay following the manufacturer's instructions (Sigma-Aldrich, St. Louis, MO, 22 USA). Based on protein, equal amounts of purified plastoglobules from WT and *fbn4* KD apple trees were used for lipid extraction. Retinol (Sigma-Aldrich, St. Louis, MO, 22 USA) was added to the purified plastoglobules prior to extraction. The plastoglobule samples were then briefly sonicated (10 s pulse), mixed with equal volumes of hexanes, vortexed vigorously, and then centrifuged at 10,000 g for 1 min. The hexanes phase was removed and dried under nitrogen. The extracted solids were dissolved in cold acetone.

### Lipid analysis by APCI LC-MS and APCI LC-MS/MS

APCI LC-MS analyses of the chloroplast and plastoglobule extracts were performed on a 3200 Q TRAP triple-quadrupole linear ion trap instrument (Applied Biosystems) in positive ion mode. A binary mobile phase gradient was delivered by a Shimadzu Prominence LC-20AD system at a flow rate of 0.4 mL/min and consisted of 0.1% v/v formic acid in methanol (solvent A), and 0.1% v/v formic acid in isopropanol (solvent B). Samples in 10 µL volume were introduced using a Shimadzu SIL-20AC autosampler and separated on a C18 column (Supelco Discovery BIO Wide Pore C18, 5 cm ×2.1 mm, 5 µm) using the following gradient: 0 min –8 min, 100% solvent A/0% solvent B; 8 min –12 min, 100%–20% solvent A/0%–80% solvent B; 12 min –16 min, 20% solvent A/80% solvent B. Instrument parameters were optimized using a direct infusion of 0.5 µg/mL retinol solution in methanol, and LC conditions were optimized using a standard mixture of β-carotene (Sigma # C4582), mono-, di-, and triacylglycerides (Sigma #1787-1AMP). The instrument conditions were as follows: ion spray voltage 5500 V; source temperature 300°C; nebulizing current 5 µA; declustering potential 20 V; entrance potential 10 V; CAD gas pressure setting “high”; collision energy 40 V with collision energy spread of 10 V.

### Retinol analysis by LC-MS

Retinol, which gives a prominent ion at *m/z* 285 was not detected in the chloroplast and plastoglobule lipid fractions analyzed by LC-MS. Therefore, retinol was used as a normalization control. During the APCI process, retinol undergoes in-source fragmentation [39]. Both intact retinol and its fragment at *m/z* 269 exhibited the same retention time in the LC-MS MIM chromatogram. Retinol was added to the chloroplasts and the plastoglobules in known quantities prior to the extraction of lipids as a normalization control.
